# British Container Breeding Mosquitoes: The Impact of Urbanisation and Climate Change on Community Composition and Phenology

**DOI:** 10.1371/journal.pone.0095325

**Published:** 2014-04-23

**Authors:** Susannah Townroe, Amanda Callaghan

**Affiliations:** Environmental and Evolutionary Biology, School of Biological Sciences, University of Reading, Reading, England; University of Western Ontario, Canada

## Abstract

The proliferation of artificial container habitats in urban areas has benefitted urban adaptable mosquito species globally. In areas where mosquitoes transmit viruses and parasites, it can promote vector population productivity and fuel mosquito-borne disease outbreaks. In Britain, storage of water in garden water butts is increasing, potentially expanding mosquito larval habitats and influencing population dynamics and mosquito-human contact. Here we show that the community composition, abundance and phenology of mosquitoes breeding in experimental water butt containers were influenced by urbanisation. Mosquitoes in urban containers were less species-rich but present in significantly higher densities (100.4±21.3) per container than those in rural containers (77.7±15.1). Urban containers were dominated by *Culex pipiens* (a potential vector of West Nile Virus [WNV]) and appear to be increasingly exploited by *Anopheles plumbeus* (a human-biting potential WNV and malaria vector). *Culex* phenology was influenced by urban land use type, with peaks in larval abundances occurring earlier in urban than rural containers. Among other factors, this was associated with an urban heat island effect which raised urban air and water temperatures by 0.9°C and 1.2°C respectively. Further increases in domestic water storage, particularly in urban areas, in combination with climate changes will likely alter mosquito population dynamics in the UK.

## Introduction

Urbanisation is a major cause of modification to the landscape. The UK continues to experience urban expansion with 80% of the population in England living in urban areas [1]. Urbanisation alters the physical environment in a way which is known to alter habitat types, species numbers and the community composition of ecosystems [2], [3]. Urbanisation also influences localised temperature dynamics (urban heat islands, UHI) through the absorption and release of solar energy trapped in buildings and through heat produced as a by-product of industry.

Mosquito species in the genera *Anopheles, Aedes* and *Culex* that have wide ranging or suitable environmental tolerances have benefitted from anthropological environmental change and can thrive in urban areas [4]. Exploitation of human environments has allowed species such as *Ae. albopictus* to dramatically increase its range globally with recent range expansion across Europe [5]. A close association of mosquitoes and humans in urban areas also facilitates mosquito-borne human disease outbreaks in other parts of the world [5], [6]. A recent example of this has been the wave of WNV outbreaks across parts of the USA vectored by *Culex* species [5], [7].

Within the UK, containers that harvest and store rainwater for domestic use (water butts) are becoming increasingly common in residential gardens; driven in part by changing climate and weather patterns that are increasing pressure on water resources. Water butts are wooden or plastic cylindrical containers that collect water from gutters. A severe drought and subsequent hosepipe ban in the spring of 2012 across Southern and Eastern England led to reports of hugely inflated sales of water butt containers [8]. The wet summer that followed is likely to have filled these containers, representing potential new habitats for mosquitoes. Water butts collect rain from roof guttering along with vegetation, animal detritus and heterotrophic microorganisms (i.e., bacteria, fungi, protozoans), providing both a habitat and food resource for mosquito larvae [9], [10]. They are designed to store water and their height precludes colonization by vertebrate and some invertebrate predators of mosquito larvae (e.g. fish, amphibians, dragonflies) [11]. Although each water butt only provides 50-250 litres of water, when combined, containers may represent a large area of habitat and can create a network of easily accessible prime mosquito larval habitat [12]. This, in combination with the UHI effect, which can increase the temperature in large UK cities by as much as 8.9°C compared to surrounding rural areas [13], may favour increased mosquito larval production in urban habitats.

In the UK, several mosquito species are known to use water butt habitats, including *Culex pipiens* L., *Culex torrentium, Culiseta annulata* and *Anopheles plumbeus* [14]. Whilst it is predicted that an increase in container habitats in the UK will increase mosquito abundance [14], there are limited data on the species composition, abundances and seasonal variation of mosquitoes using urban and rural habitats. Mosquito species which are a human biting nuisance or are potential vectors of human or wildlife diseases such as WNV or avian pox virus (Poxviridae) are potentially going to thrive in urban environments where they will come into close contact with humans and birds.

The focus of this study is the standard garden water butt container and the species composition and phenology of the mosquitoes that breed in them in the South East of England. We investigated the wider habitat matrix effect, and tested the hypothesis that containers in urban type landscapes would support higher larval densities but lower species richness than containers in rural landscapes. We examined mosquito colonisation and phenology to establish seasonal activity of species in the two different landscape types. We discuss how urbanisation of the UK landscape influences various aspects of mosquito biology and the implications of future land use and climate change scenarios on mosquito population dynamics.

## Material and Methods

### (a) Study sites and experimental containers

The study was undertaken in Berkshire and Oxfordshire, England. Two land use types were selected for the study; urban residential gardens and rural residential gardens, with 10 sites of each in 2011 and eight sites of each in 2012. Sites in the urban land use type were located in the town of Reading within 4 km of the town centre (51”27′19.99”N, 0”58′17.89”W, total town area 55.35 km^2^, human population 232,662 [15]). Sites in the rural land use type were located in gardens in villages, hamlets or isolated dwellings where settlement types were sparse and of a distance greater than 1 km from suburb limits. The study was advertised in local media and members of the public volunteered use of their gardens. The first 10 urban and 10 rural sites which fit the criteria were selected for use in the study. We would like to thank volunteering members of the public for allowing access to their gardens for sampling.

An 80 litre black plastic dustbin (44.5×58.5 cm) acting as a water butt (without gutter downpipes) was placed at each site. All containers were positioned against an outside wall of a building facing North, North East or North West and/or in shade for more than 50% of the day to control for exposure to solar radiation. Each container was filled with 50 litres of tap water, 8.7 g of dried and coarsely ground laboratory Coleoptera specimens and 300 g of partially decomposed leaf litter from mixed deciduous trees from a single local source. This type of low-to-medium detrital load including both animal detritus and mixed leaf litter provides a suitable and natural source of nutrition for developing mosquito larvae [16]. Each container was fitted with a secure lid incorporating four 4 cm diameter holes to allow invertebrates to enter. All containers remained in position for eight months.

### (b) Sample collection

Containers were sampled weekly for 29 weeks from 7^th^ April 2011. In March 2012, 16 new containers were set up at 8 urban and 8 rural sites following the same method as the previous year. These containers were sampled weekly for 30 weeks from 4th April 2012.

Sampling was carried out using a device adapted from Onyeka [17]. The device included three sections of drain pipe (4 cm high, 0.4 cm thick and 8 cm diameter) bolted together in line with fine mesh net glued to the bottom of each ring and a flexible wire handle attached to the outer edge of the furthest two rings. The device was lowered into the container and allowed to rest on the bottom for 5 minutes before being drawn swiftly up through the water to collect animals. This method was carried out once per container per sampling event and the depth of water in each container was recorded at this time. Mosquito larval abundance, instar and subfamily and pupal abundance were recorded following collection with the sampling device. All larvae were replaced in the container to prevent interference of removal sampling. All pupae collected in the sample device were not returned. Additional pupae were collected from the water surface with a pipette during a 10 minute search period at each sampling event. All pupae were taken to the laboratory for rearing to adult then frozen at −22°C. Adults were identified using a 10–40× magnification microscope using the key of Cranston et al. [18]. Slide preparations were assembled for male terminalia of *Cx pipiens* and *Cx torrentium* for differentiation between species [19].

Container water temperature and adjacent air temperature was recorded weekly at all sites during sampling in 2011. During 2012, air and water temperature data were collected from four randomly selected sites in both landscape types using data loggers (Tiny Tag Talk 2 Temperature Logger, Gemini Data Logger UK ltd, Chichester, West Sussex, UK). At each selected site one data logger was attached to a stake and secured in the ground next to the container, out of direct sunlight and with a waterproof canopy fitted, to record air temperature. One data logger measured water temperature within the container using a water-resistant probe submerged at a depth of approximately 20 cm at the edge of the container. Data loggers recording air temperature took measurements of average temperature every 30 minutes from 24/3/12 to 9/11/12. Data loggers recording water temperature took measurements of average, maximum and minimum water temperatures every 30 minutes from 2/4/12 to 9/11/12.

### (c) Statistical analysis

Mean mosquito abundance data per container were used for analyses. First instar larvae were excluded from analyses as they were too small to detect reliably. The sampling period was divided equally to provide three seasons that were defined as early season (4^th^ April- 7^th^ June), mid-season (14^th^ June- 17^th^ August), and late season (23^rd^ August- 25^th^ October). The relationship between mean mosquito abundance and location, year and season were analysed using generalised linear mixed-effects models with ‘location’, ‘year’ and ‘season’ as fixed effects and ‘site’ as random effect. Model fitting and estimates were obtained with the linear mixed-effects (lmer) package lme4 in R version 2.15.2 [20] and fit by Laplace approximation with a specified ‘poisson’ error family. We followed Crawley [21] and used the ‘anova’ function in the lme4 package to compare the quality of fit between models. Model fit and the significance of including the varying slope parameters were tested using AIC (Akaike's Information Criterion) and log-likelihood ratio statistics (LLR λ2) [22]. Wald's Z statistic and probability ‘P’ values of best fit models are quoted throughout. Degrees of freedom are not quoted since there is currently no consensus on the correct method for their calculation in lmer models [22], [23].

Temperature data were averaged over each year with averaged values for each site used for analyses. The relationship between air and water temperature and the relationships between temperature and location and year were analysed using linear models in R version 2.15.2 [20] with model fit assessed by the distribution of residuals. Means and standard errors are reported throughout, and significance was assigned at the 5% level.

## Results

### (a) Mosquito population dynamics

**Figure 1:**
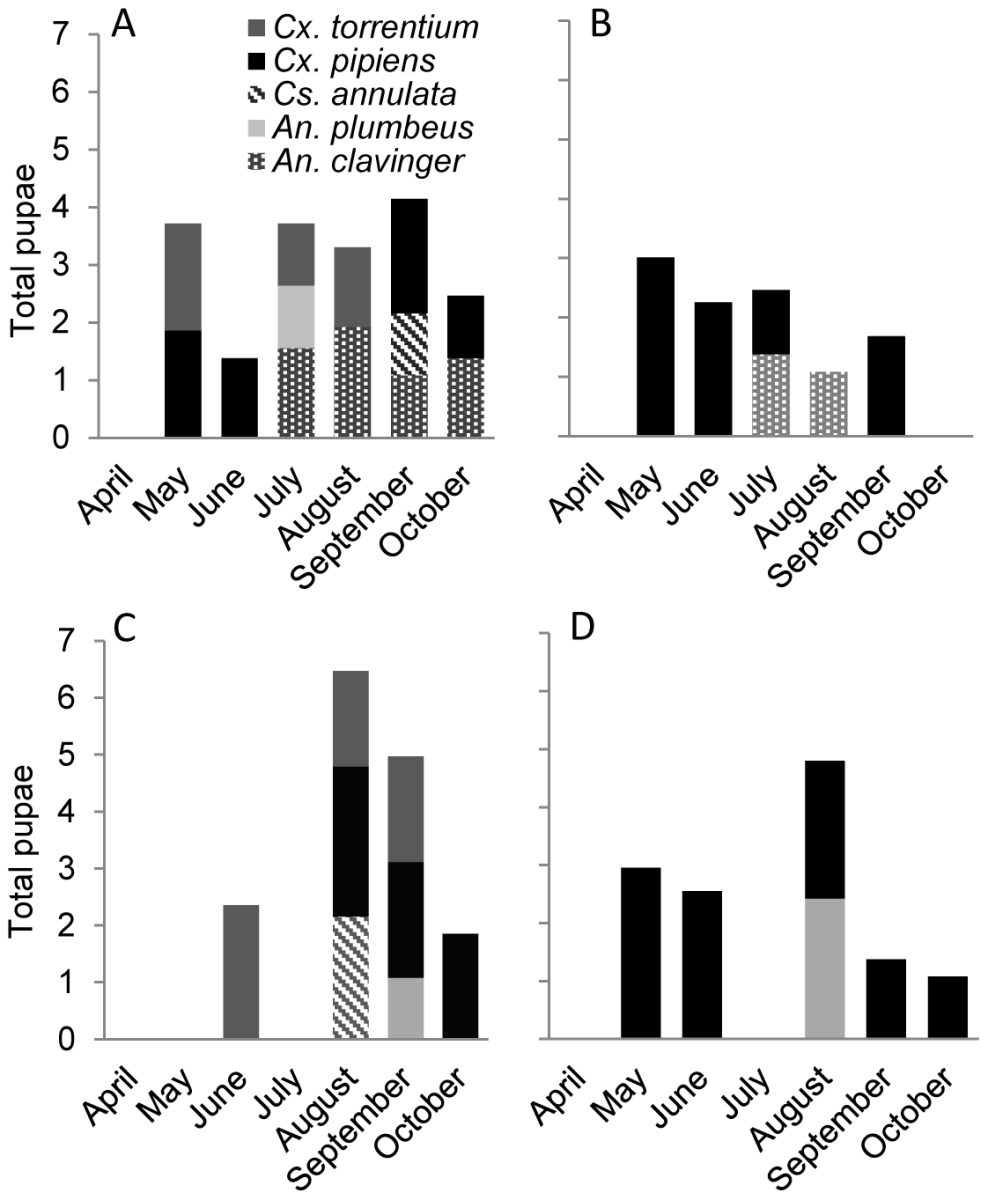
Total number and composition of mosquito species collected from containers. Individuals collected as pupae in 2011 from rural (a) and urban (b) containers and in 2012 from rural (c) and urban containers (d), April to October. Note the Y axis is log10 transformed.

Throughout 2011–2012, five mosquito species were recorded breeding in experimental garden water butt containers (Table 1, figure 1). *Culex pipiens* was the most abundant species (77% of all pupae collected). In urban containers only 3 species were recorded; *Cx pipiens* (both years), *An. clavinger* (2011) and *An. plumbeus* (2012). *Cx pipiens* dominated urban containers during both years representing 97% of the community in 2011 and 85% in 2012. In 2011 the rural containers held five mosquito species (Table 1, figure 1) although *An. clavinger* was not recorded in 2012. *Cx torrentium* was found breeding in 25% (2011) and 30% (2012) of rural containers but was absent from the urban sites. Figure 1 shows that no mosquito pupae were found during July 2012 in either urban or rural locations.

**Table 1:** Total (%) of mosquito species collected (as pupae) from rural and urban containers in 2011and 2012 and their potential as vectors of disease.

	2011			2012			2011 & 2012	Anthrop-ophagic*	Likely bridge vector*	Vector of disease in Europe*
Species	Rural	Urban	Total	Rural	Urban	Total	Total			
*An. clavinger*	144 (31)	36 (3)	180 (10)	0 (0)	0 (0)	0 (0)	180 (4)	X	-	Batai Virus
*An. plumbeus*	12 (3)	0 (0)	12 (1)	12 (1)	264 (15)	276 (9)	288 (6)	X	X	WNV, Malaria (*P. vivax* and *P. falciparum*)^1^
*Cx pipiens*	192 (41)	1212 (97)	1404 (82)	612 (55)	1536 (85)	2148 (74)	3552 (77)	-	-	Ockelbo virus, SINV, TAHV, USUV, WNV
*Cx torrentium*	108 (23)	0 (0)	108 (6)	348 (31)	0 (0)	348 (12)	456 (10)	-	-	Ockelbo Virus, SINV
*Cs. annulata*	12 (3)	0 (0)	12 (1)	144 (13)	0 (0)	144 (5)	156 (3)	X	X	TAHV, USUV
Total	468	1248	1716	1116	1800	2916	4632			

*Information of host preference taken from Medlock et al. [32] and disease vector status from Medlock et al. [31] and ^1^Schaffner et al. [28].

Culicine larvae and pupae and Anopheline pupae were more abundant in urban compared with rural containers (Table 2 & 3), although the opposite was true for Anopheline larvae (Table 2). All mosquito larvae were more abundant in 2011 than 2012 (Table 2 and 3). There was a strong interaction effect of location and year on larvae from both subfamilies combined and on culicine larvae alone, with significantly higher larval abundances in the urban location in 2011 (Z = 5.085, P<0.001; Z = 5.006, P<0.001, respectively). Pupae of both subfamilies were marginally more abundant in 2012 than 2011, however this was not significant.

**Table 2:** Mean total mosquito densities per container (±se) of each development stage of subfamilies Anophelinae and Culicinae by location and year.

	2011	2012	Rural	Urban
**Anophelinae**				
Total larvae	5±1.4	2.8±1.3	4.7±1.6	3.4±1.1
Pupae	0.4±0.1	0.6±0.3	0.3±0.1	0.6±0.2
**Culicinae**				
Total larvae	99.9±20.8	66.2±13.7	73.0±15.3	97.0±21.4
Pupae	6.0±2.0	8.9±3.6	4.4±1.8	10.2±3.4
**Subfamilies combined**				
Total larvae	105±20.6	69±13.8	77.7±15.1	100.4±21.3
Pupae	6.4±2.0	9.5±3.7	4.7±1.8	10.8±3.4

Results from best fit generalised linear mixed model fit by Laplace approximation. There was no significant main or interaction effect of ‘location’, ‘year’, or ‘season’ on *Anopheles* (analyses not shown). Bold type denotes significance at the 5% level.

**Table 3:** Main effects and interaction effects of ‘location’ and ‘year’ on mean total of all mosquitoes and of Culicinae and main and interaction effects of ‘season’ and ‘location’ on Culicinae mosquitoes.

	Pupae		Total larvae	
Factor	*Z*	*P*	*Z*	*P*
**Location and year**				
**Subfamilies combined**				
Location urban	2.195	**<0.05**	3.354	**<0.001**
Year 2012	−0.03	0.9	−12.335	**<0.001**
Loc.urban x year2012	0.03	0.9	5.085	**<0.001**
Culicinae				
Location urban	2.22	**<0.05**	11.054	**<0.001**
Year 2012	−0.03	0.9	−12.301	**<0.001**
Loc.urban x year2012	0.03	0.9	5.006	**<0.001**
**Season and location**				
Culicinae				
Mid-season vs Early season	−4.109	**<0.001**	19.816	**<0.001**
Late season vs Early season	−0.84	0.4	−4.879	**<0.001**
Loc. urban x mid-season	−2.149	**<0.05**	−26.076	**<0.001**
Loc. urban x late season	−7.525	**<0.001**	−20.785	**<0.001**

### (b) Container colonisation and seasonal niche separation

Anopheline mosquitoes colonised nine rural and six urban containers in 2011 with larvae present at both site types from the 30^th^ of June until the final week of sampling. Colonisation rates were lower in 2012 with *Anopheles* present in only half the containers in each site type; from 5^th^ July to 12^th^ September in urban containers and 19^th^ July until the final week of sampling in rural containers. There were no significant differences in Anopheline larvae abundance between seasons.

**Figure 2:**
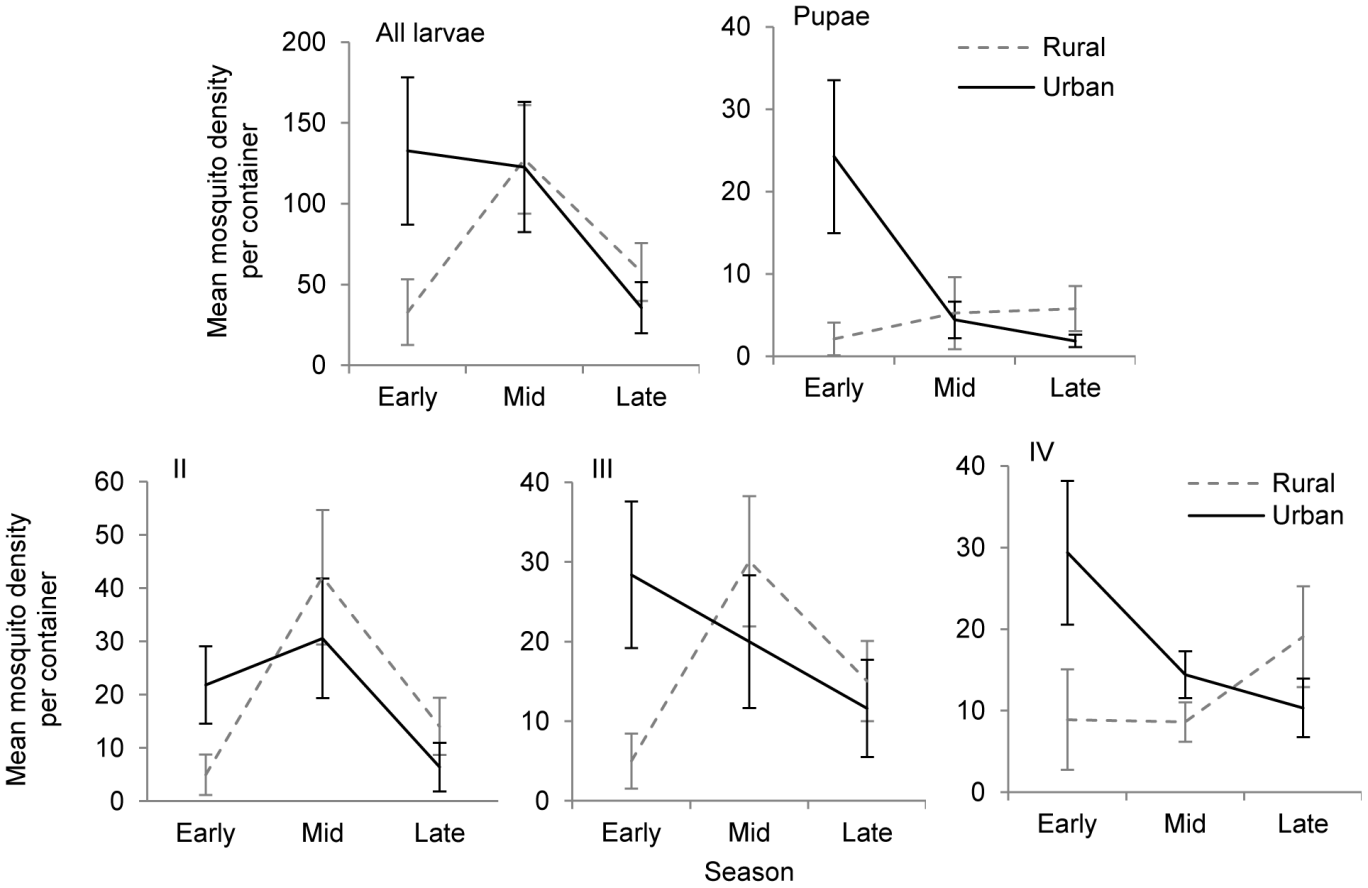
Interaction effects of ‘season’ and ‘location’ on culicine larval densities. Interaction effects of ‘season’ (x axis) and ‘location’ (family of lines) on mean total culicine larval densities (±se) per container and mean total densities of each immature development stage, II-IV instar larvae and pupae (y axis), April to October 2011 and 2012.

Culicinae larvae were present in containers in both landscape types from 12^th^ April until 20th October in rural and until 27th October in urban containers in 2011. Whilst in 2012 Culicinae larvae were present in urban containers from 18^th^ April and considerably later in rural containers, from 7^th^ June. Larvae were present at both site types until 25^th^ October 2012. A greater number of containers were colonised by Culicinae larvae at the beginning of the season in urban compared to rural gardens. Analysis of mean Culicinae larval abundance across the three seasons ‘early’, ‘mid’ and ‘late’ showed that overall abundances were highest mid-season (Table 3, Fig. 2). However, there was an interaction effect of location and season with significantly higher abundances of all Culicinae development stages in the early season in urban containers compared to rural containers (Table 3, Fig. 2).

### (c) Urban Heat Island (UHI) effect

A significant Urban Heat Island (UHI) effect was detected in Reading over both years with air temperatures on average 0.9°C higher at urban sites and water temperatures on average 1.3°C higher at urban sites than rural (Table 4). The relationship between air and water temperatures was highly significant (adjusted R^2^ = 0.83, *F*_(1, 26) = _133.3, *P*<0.001) (see figure S1). There was a significant difference in temperatures between years with both air and water temperatures on average lower in 2012 than 2011 (Table 4). In 2011 average temperatures followed an expected pattern and rose gradually with each successive month, from 14.6°C in April to a peak of 17.9°C in July and then gradually declined again to 13°C in October. 2012 did not follow the same pattern and instead average temperature initially peaked in May (15.8°C) followed by a cooler June (14°C) and July (15.6°C) and then another peak in August (17.2°C). Importantly, there was a very large difference in the minimum temperatures in July in 2011 (15°C) and 2012 (5.7°C).

**Table 4:** Mean (±se), maximum and minimum air and water temperatures and effects of year and location on temperatures in 2011 and 2012.

Factor	Temperature (°C)		d.f. effect, error	Test statistic	*P*
	**2011**	**2012**			
Air	15.9±0.17	13.1±0.26	2,25	129.25	**<0.001**
Water	15.6±0.18	13.6±0.26	2,25	63.25	**<0.001**
	**Urban**	**Rural**			
Air	15.6±0.38	14.7±0.39	2,25	16.13	**<0.001**
Water	15.7±0.24	14.4±0.33	2,25	28.9	**<0.001**
**2011**					
**Air**	16.4±0.16	15.5±0.21	1,18	3.49	**0.002**
Max.	22.9	28			
Min.	8.1	7.1			
**Water**	16.1±0.17	15.08±0.23	1,18	3.54	**0.002**
Max.	25.2	23.2			
Min.	8.6	8			
**2012**					
**Air**	13.5±0.31	12.7±0.43	1,18	1.84	0.1
Max.	34.9	29.9			
Min.	−0.1	−2.7			
**Water**	14.4±0.06	12.8±0.27	1,18	5.69	**0.001**
Max.	29.6	24.5			
Av. Max.	16.5±0.23	14.3±0.42	1,18	4.61	**<0.01**
Min.	3.3	1.6			
Av. Min.	12.8±0.07	11.6±0.2	1,18	5.06	**<0.01**

In 2011 the UHI effect was significant for both air temperature (0.9°C) and water temperature (1.1°C). In 2012, the average air temperature UHI effect was 0.8°C (ns). The average water temperature UHI effect was 1.6°C with an average maximum effect of 2.2°C and average minimum effect of 1.2°C. Water temperatures were significantly higher at urban sites for all three measurements taken; average, maximum, and minimum (table 4). The difference between maximum urban and rural air temperatures reached 5°C and water temperatures reached 5.1°C.

## Discussion

The results of this study show that artificial freshwater habitats such as garden water butts support diverse mosquito populations, with significantly higher larval densities but reduced species richness in urban gardens compared to rural gardens. The broad indications of this study support the notion that urbanisation of the landscape influences various aspects of mosquito biology including the diversity and density of mosquito species and the seasonality of peak larval abundances.

The species recorded in this study are all common and widespread in the UK and known to utilise artificial container habitats [18]. The variation found in species distribution in both landscape types may be explained by the likely variation in aquatic habitat diversity in each landscape type. Rural areas are likely to contain a greater diversity of natural larval habitats that support more species-rich and diverse populations which can utilise but not necessarily rely on containers for breeding [24]. In contrast, historical development and continued alteration of urban Reading has probably reduced the abundance and diversity of natural type larval habitats and replaced them with artificial larval habitats. The widespread presence of *Cx pipiens* was expected since this is one of the commonest British mosquitoes and its presence in artificial container habitats, along with its sympatric sibling species *Cx torrentium,* has been well documented in the UK [24], [25]. It is notable that *Cx torrentium*, which is similar in many respects to *Cx. pipiens*, was widely distributed across rural sites but absent from urban sites. Previous studies report *Cx torrentium* predominating over *Cx pipiens* in peri-domestic habitats [25]. However our results concur with more recent survey work that failed to find *Cx torrentium* in urban areas [24], suggesting that this species is either unable to colonise or to survive in containers in urbanised areas. Anophelinae species exploited the container habitats in both landscape types, although these do not represent their main breeding habitat which is more commonly tree holes for *An. plumbeus* and shaded ponds, ditches, and streams, for *An. clavinger* [26]. These natural habitat types are likely to be more abundant in rural areas supporting larger populations of Anopheles here, in part explaining why we found greater abundances of Anophelinae larvae in rural containers. The relatively high abundance of *An. plumbeus* during the late season of 2012, and dramatic increase in abundance from the previous year is notable. Early surveys of *An. plumbeus* in the UK describe containers as occasional breeding sites [27]. However, the numbers found in this study indicate successful use of container habitats and imply a similar trend to that seen in Continental Europe where *An. plumbeus* is shifting habitats from almost exclusively breeding in tree holes to exploiting a wider array of novel man-made larval breeding habitats, including water butts [28]. Population expansions or increased use of human associated habitats by *An. plumbeus* is of particular human health importance because this species is a day active and vicious human biting mosquito. It is a candidate bridge vector of WNV and infectivity tests of European strains have revealed that it can transmit *Plasmodium falciparum*, confirming its competence as a bridge vector of tropical malaria [28].

Containers in urban gardens held significantly greater densities of larvae and pupae than containers in rural gardens. This is largely due to the dominance of *Cx pipiens* which has a high ecological and physiological flexibility [3] allowing exploitation of the urban environment where lower mosquito species richness provides reduced intraspecific competition. In countries where *Culex* species transmit diseases, their domination in urban environments supports disease outbreaks [29], [30]. In the UK, *Cx pipiens* is ornithophilic and not likely to cause any biting nuisance [31]. The British Isles are currently free from mosquito-borne disease transmission to humans [32]. However, there is evidence of WNV neutralising antibodies in British resident birds [33] and, as a known competent WNV vector, *Cx pipiens* is a likely candidate for enzootic transmission of this disease among birds where circumstances are conducive and particularly in urban areas where they dominate in the UK [32].

In Reading a significant average air temperature UHI effect of 0.9°C was detected over the whole study period. In 2012 average water temperature differences reached 2.2°C with maximum differences as high as 5°C. The biological importance of a 1°C air temperature increase to *Cx pipiens* is unclear. However, warmer air temperatures are known to affect adult flight activity, digestion of blood meal and egg development by females [9]. Increases in water temperature of 5°C can influence larval development times, speeding up *Cx pipiens* development by 8.3 days [34]. Our results show that when comparing larval abundances between 2011 and 2012, abundances were significantly greater during 2011 when temperatures were significantly higher than the following year. Conversely, low temperatures may retard larval development. Our results show that during July of 2012 far fewer than expected pupae were collected. This coincided with unusually low average air temperatures for this month and minimum air temperatures of only 5.7°C (9°C lower than minimum temperatures in July of the previous year), suggesting a link between air temperature and the number of mosquitoes potentially emerging.

Our results showed that *Culex* mosquitoes utilised an earlier seasonal niche in the urban environment. Termination of diapause and initiation of gonotrophic activity is influenced by increasing environmental temperature [17], [35], with warmer temperatures in the antecedent winter season and early spring correlating positively with increased spring *Culex* abundance [36]. Higher temperatures in urban areas may extend the breeding season, increasing the rate of reproductive cycling and number of host interactions. Therefore climate change and UHI effects can alter the phenology of *Culex* mosquitoes leading to early exodus from hibernation sites, increased abundances of females [35], [37] and, in countries where they transmit disease, early season outbreaks of disease [38]. Early spring abundances of *Cx pipiens* at urban sites may also be synchronised with early breeding urban birds to exploit the increased susceptibility at this time of brooding parents and nestlings to mosquito attack [39].

In conclusion, urbanisation changes the environment in a way which has pronounced consequences to British mosquito communities. The changing climate and weather patterns that encourage the human activity of domestic garden water storage also result in an abundance of container breeding habitats, particularly in urban areas. Conditions in the urban environment appear to restrict colonisation to only those species with suitable environmental tolerances, potentially facilitating a shift in the habitat niche of *An. plumbeus* and allowing *Cx pipiens* populations to dominate and thrive. The abundance of these water resources, and synanthropic birds as potential food sources and an UHI effect which maintains warmer air and water temperatures appears to be driving the phenology of *Culex* mosquitoes in urban areas to become earlier than those in rural areas. These changes are conducive to mosquito populations becoming more of a nuisance to humans and an increase in potential for transmission of human and wildlife diseases. Further increases in domestic water storage in combination with climate changes will likely alter mosquito population dynamics.

## Supporting Information

Figure S1 **Average air and water temperatures.** Average weekly air and water temperatures in 2011 (a) and average daily air and water temperatures in 2012 (b) at rural and urban sites.
(TIFF)
